# *Mahuang-Fuzi-Xixin* Decoction Reverses Depression-Like Behavior in LPS-Induced Mice by Regulating NLRP3 Inflammasome and Neurogenesis

**DOI:** 10.1155/2019/1571392

**Published:** 2019-11-11

**Authors:** Wen Jing, Shanshan Song, Hong Sun, Ying Chen, Quan Zhao, You Zhang, Guoliang Dai, Wenzheng Ju

**Affiliations:** ^1^Affiliated Hospital of Nanjing University of Chinese Medicine, Nanjing 210029, China; ^2^Department of Clinical Pharmacology, Affiliated Hospital of Nanjing University of Chinese Medicine, Nanjing 210029, China

## Abstract

Evidence suggests that inflammation and neurogenesis play an important role in major depressive disorder (MDD). *Mahuang-Fuzi-Xixin* decoction (MFX), as the traditional Chinese prescription, has been widely applied for asthma, migraine, and MDD in clinics. However, the effects of MFX on the potential mechanism in MDD are still unclear. Hence, the present study is aimed at exploring whether the antidepressive effect of MFX is connected to the anti-inflammatory and promoting neurogenesis. Besides, lipopolysaccharide (LPS) of Gram-negative bacteria can induce depressive-like behaviors. We demonstrated that administration of MFX corrected the depressive-like behaviors in LPS-induced mice and significantly decreased the expression of IL-1*β* in the hippocampus. LPS injection induced a significant increase in the levels of NLRP3, cleaved caspase-1 p20, and ASC in the hippocampus, as well as Trx-interacting protein (TXNIP), and MFX could reverse this change. What is more, treatment of MFX increased the level of doublecortin (DCX), brain-derived neurotrophic factor (BDNF), and tropomyosin-related kinase receptor B (TrkB) in the hippocampus which means that MFX could promote the neurogenesis. In conclusion, the study indicates that MFX relieves a depressive-like state in LPS-induced mice through the inhibition of the NLRP3 inflammasome and the enhancement of the neurogenesis pathway.

## 1. Introduction

Major depressive disorder (MDD), characterized by mood despondency and anhedonia [1], is one of the main causes of the disability and high mortality rate worldwide [2–5]. However, current antidepressants used in clinic cannot meet the needs with respect to both efficacy and severe side effects; besides, 30% to 50% of patients are not sensitive to these antidepressants [6]. Therefore, there is still an urgent need to find drugs which would be safe and effective.

It has been known for decades that depression is closely associated with inflammation [7] since Maes proposed in 1995 [8]. Moreover, the American Psychiatric Association included inflammatory markers in the guidelines for depression diagnosis in 2013 [9]. Proinflammatory cytokine interleukin 1 beta (IL-1*β*) is demonstrated to participate in inflammatory responses in the central or peripheral nervous system in MDD [10]. The maturation of pro-IL-1*β* depends on the IL-1*β*-converting enzyme, which is an important component of inflammasome. Inflammasome is a multiple protein complex in the assembly of intracytoplasmic pattern recognition receptors (PRRs). Studies indicate that the activation of inflammasome nucleotide-binding oligomerization domain-like receptor family pyrin domain containing 3 (NLRP3) promotes the proinflammatory cytokine secretion, including IL-1*β* and IL-18 [11–16], and then reduced to a series of inflammatory reactions. Studies show that the NLRP3 inflammasome in blood cells of patients with MDD was activated, and the increased serum levels of IL-1*β* and IL-18 were positively correlated with Beck Depression Inventory (BDI) scores [17]. Then, the NLRP3 inflammasome is considered as a new promising target for the treatment of MDD [18–21]. More importantly, the Trx-interacting protein (TXNIP) plays an indispensable role in the activation of NLRP3 inflammasome [22].

Additionally, neurogenesis has been implicated in the pathogenesis of MDD [23]. Neurogenesis, specifically in the dentate gyrus (DG) of the adult hippocampus, gives rise to new neurons throughout life. Decreased neurogenesis could lead to a smaller hippocampus, consistent with this phenomenon, patients with depression had decreased hippocampal volume [24–26]. Besides, studies show that decreased neurogenesis is associated with lowered levels of neurotrophins, like brain-derived neurotrophic factor (BDNF) [27, 28]. Intriguingly, studies show that the activation of NLRP3 inflammasome in the cortex, hippocampus, or amygdala was reversed in neuroligin3 (NLGN3) knockout mice; the BDNF contents were restored by NLGN3 deficiency [29]. Therefore, the decreased BDNF release induced by the activated NLRP3 inflammasome was a key pathological mechanism of the depressive behaviors induced by sleep deprivation [30]. Correspondingly, tropomyosin-related kinase receptor B (TrkB), as the high affinitive BDNF receptor, is required for induced proliferation and neurogenesis by antidepressants and voluntary exercise [31].

In recent years, traditional Chinese medicine (TCM) has been well recognized in alleviating symptoms of depression for safety and effectiveness [32–34]. *Mahuang-Fuzi-Xixin* decoction (MFX) was first prescribed in Treatise on Febrile Diseases and has efficiency in the treatment of migraine, asthma, rheumatoid arthritis, and MDD [35–38]. Studies show that MFX has good anti-inflammatory and immunosuppressive effect, as well as antioxidant effect [39, 40], which may be related to its clinical antidepressive effect. MFX composed of *Radix Aconiti Lateralis*, *Ephedrae*, and *Asarum* were mixed at the ratio of 3 : 2 : 1. *Radix Aconiti Lateralis* polysaccharide and alkaloids, which are the virtual components, have pharmacological action in anti-inflammation, antidepression, antiepileptic, and analgesic [41–44]. *Ephedrae* alkaloids such as ephedrine and pseudoephedrine are the main constituents and have the effect on antiallergic activity, anti-influenza virus, and so on [45, 46]. The main active ingredients of *Asarum* are the essential oils, asatone, and asarinin. *Asarum* is effective on anti-inflammation and analgesia [47, 48]. Although there is increasing evidence for MFX's therapeutic benefits for depression-like behaviors in preclinical studies, little is known about the underlying therapeutic mechanism. In light of this, we wonder the antidepressant efficacy of MFX on LPS-induced mice and linked this effect to the increased expression of BDNF by the inhibited NLRP3 inflammasome. Our results suggest that MFX has antidepressant effect in LPS-treated mice and MFX significantly inhibits the activation of NLPR3 inflammasome, decreases the proinflammatory cytokines, and boosts neurogenesis in LPS-induced mice.

## 2. Materials and Methods

### 2.1. Reagents

The makers for the quality control of MFX with ephedrine hydrochloride and pseudoephedrine hydrochloride were purchased from National Institutes for Food and Drug Control (Beijing, China), and benzoylaconine, benzoylmesaconine, benzoylhypaconine, and asarinin were purchased from Chengdu Mansite Pharmaceutical Co., Ltd. (Sichuan, China). Escitalopram was purchased from Dalian Meilun Biotechnology Co., Ltd. (Liaoning, China). Lipopolysaccharide (LPS) was bought from Sigma-Aldrich Co., Ltd. (St. Louis, USA). Rabbit polyclonal anti-NLRP3 (ab214185), rabbit polyclonal anti-IL-1*β* (ab9722), rabbit monoclonal anti-TXNIP (ab188865), rabbit monoclonal anti-DCX (ab207175), rabbit polyclonal anti-DCX (ab18723), and rabbit monoclonal anti-BDNF (ab108319) were acquired from Abcam. Rabbit monoclonal anti-ASC (67824S) and rabbit monoclonal anti-TrkB (4603T) were offered by Cell Signaling Technology. Mouse monoclonal anti-caspase-1 (sc-56036) was obtained from Santa Cruz Biotechnology. Goat polyclonal anti-IL-1*β* (AF-401-NA) was purchased from R&D. Rabbit polyclonal anti-*β*-tubulin (abs131994) and rabbit polyclonal anti-GAPDH (abs132004) were obtained from Absin Bioscience Inc. Alexa Fluor 488 Goat anti-Rabbit IgG (A-11034) and Alexa Fluor 594 Rabbit anti-Goat IgG (A27016) were purchased from Invitrogen.

### 2.2. Preparation of MFX

MFX consisted of three Chinese herbs as the following: *Radix Aconiti Lateralis* (voucher specimen no. 1609002), *Ephedrae* (voucher specimen no. 171001), and *Asarum* (voucher specimen no. 160810002). They were purchased from Tongrentang Chinese Pharmaceutical Co. Ltd. (Beijing, China), identified by Dr. Qian Zhang, School of Pharmacy, Nanjing University of Chinese Medicine. *Radix Aconiti Lateralis*, *Ephedrae*, and *Asarum* were mixed at the ratio of 3 : 2 : 1 (*w*/*w*, with a total weight of 180 g). For extraction, according to the relative literature [49, 50], combined with the purpose of this experiment, the method of “one dose and two decoctions,” which is commonly used in the clinical practice was selected. The raw herbs were soaked with 8-fold of water (*v*/*w*) for 30 min, then followed by heating extraction at 100°C for 2 h. Subsequent extractions were carried out with 6-fold of water (*v*/*w*) for another 1.5 h. The supernatant was combined and concentrated under reduced pressure at a temperature below 60°C. The yielding product weighted about 72.56 g. The aqueous extracts were stored at -20°C before use. The yield of aqueous extract was about 40.3% (*w*/*w*).

### 2.3. The Quality and Constitutes of MFX Aqueous Extract

The determinations of ephedrine hydrochloride, pseudoephedrine hydrochloride, benzoylmesaconine, benzoylaconine, benzoylhypaconine, and asarinin in the extract of MFX were analyzed by HPLC-DAD. The analysis was performed on an Agilent 1100 HPLC system and Agilent ZORBAX SB-C18 column (4.6 mm × 250 mm, 5 *μ*m), with a flow rate of 1.0 mL/min, column temperature of 35°C, and injection volume of 10 *μ*L. The detection wavelength of ephedrine hydrochloride and pseudoephedrine hydrochloride was 210 nm, and benzoylmesaconine, benzoylaconine, benzoylhypaconine, and asarinin were detected at 235 nm wavelength. The mobile phase of ephedrine hydrochloride and pseudoephedrine hydrochloride was composed of A (acetonitrile) and B (0.1% phosphoric acid, *v*/*v*) with a gradient elution: 0–10 min, 4–6% A; 10–19 min, 6–35% A; and 19–20 min, 35–4% A. The mobile phase of benzoylmesaconine, benzoylaconine, benzoylhypaconine, and asarinin was composed of A (acetonitrile) and B (0.1% phosphoric acid, *v*/*v*) with a gradient elution: 0–8 min, 30–50% A; 8–15 min, 50–70% A; and 15–16 min, 70–30% A.

### 2.4. Animals

Adult male Balb/C mice weighing 18-20 g were purchased from the Shanghai Xipuer-Beikai Experimental Animal Co., Ltd. (Shanghai, China). Animals were kept in standardized environmental conditions (22-25°C, 12 h light/dark cycle with light on at 07:00 a.m.) with free access to food and water and housed five per cage. All animal procedures were carried out in accordance with the Guide for the Care and Use of Laboratory Animals approved by the Institutional Animal Care and Use Committee at Nanjing University of Chinese Medicine.

### 2.5. Drug Treatment

**Figure 1:**
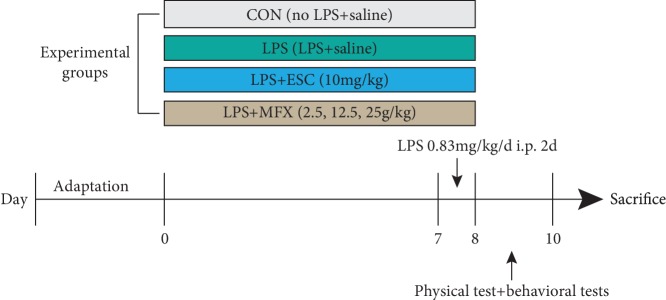
Experimental procedure.

After 1 week of acclimation, the mice were randomly assigned to six groups (*n* = 10/group): control group (CON), LPS group (LPS), LPS+escitalopram group (LPS+ESC, 10 mg/kg), and LPS+MFX groups (LSP+MFX-L, LPS+MFX-M, and LPS+MFX-H; 2.5, 12.5, and 25 g/kg). Animals in LPS+MFX groups were administered orally with *Mahuang-Xixin-Fuzi* decoction once daily for 1 week at the dose of 2.5, 12.5, or 25 g/kg, respectively, and the LPS+escitalopram group received intraperitoneal (i.p.) injections of escitalopram (10 mg/kg). In addition, except for those in the control group, the mice were injected intraperitoneally (i.p.) with LPS for two days at a dose of 0.83 mg/kg. All drugs were dissolved and diluted with 0.9% saline. In order to reduce the impact of the different administrations, the control group received the same volume of saline solution as that of drugs. All chemicals were administered in a volume of 10 mL/kg of body weight (Figure 1). The administered dose and the duration of the treatment were selected according to references [51–54], and a pilot experiment was performed in our laboratory.

### 2.6. Physical and Behavioral Tests

The physical test includes the body weight; additionally, behavioral tests include the sucrose preference experiment, open field test, tail suspension test, and forced swim test. All tests were performed between 9:00 a.m. and 17:00 in a quiet room. Behavioral testers were blinded for experimental groups.

#### 2.6.1. Sucrose Preference Test (SPT)

To establish a baseline sucrose preference, animals were given with one bottle of 2% (*w*/*v*) sucrose and one bottle of water for 3 days. To prevent possible effects of side preference in drinking behavior, the positions of bottles were switched every 12 hours. Thereafter, after 18 hours of water deprivation, every mouse was placed in a single cage with one bottle of 2% sucrose solution and one bottle of water. The positions of the bottles were switched after 1 hour. Then, water and sucrose intakes were measured 2 hours later. Sucrose solution was then removed so that only water was available, and mice were treated with drugs, followed by saline or LPS. The test phase of the experiment started 12 h after the last LPS injection and familiarization phase. Sucrose preference was calculated by the ratio of the sucrose consumption versus the total consumption of both water and sucrose.

#### 2.6.2. Open Field Test (OFT)

To examine the effects of MFX on locomotor activity in mice, the OFT was performed 24 h after the drug exposure. A well-illuminated (~300 lux) transparent acrylic cage (40 × 40 × 15 cm) was applied for the test. At the start of each trial, a mouse was gently placed in the center of an arena (40 cm × 40 cm × 40.5 cm) and left to freely explore the area for 5 minutes. The path taken of each mouse was recorded by a camera, and parameters like total crossing and time spent at the center of the open field were scored using ANY-maze software (Stoelting Co. Ltd., USA). The testing apparatus was thoroughly cleaned before each animal using 75% ethanol.

#### 2.6.3. Tail Suspension Test (TST)

TST was performed with a computerized device which allowed six mice to be tested at one time. Each individual mouse was suspended 50 cm above the floor by adhesive tape placed approximately 1 cm from the tip of the tail. The activities of the mice were videotaped, and ANY-maze software was used to calculate the time of immobility during the last 4 minutes in a 6-minute testing period.

#### 2.6.4. Forced Swim Test (FST)

FST was conducted as described previously [55]. Briefly, mice were individually placed in a cylinder (20 cm diameter, 40 cm height) containing 30 cm (depth) of water at 22–24°C and swam for 6 min. The water depth was set to prevent mice from touching the bottom with tails or hind limbs. The behaviors were recorded, and the immobile time during the last 4 minutes of the test was counted by using ANY-maze software. The mouse was judged to be immobile when it floated motionless in the water with necessary small movements to keep its head above the water. At the end of the swim test, the mice were removed from the water and dried with a hair drier.

### 2.7. Immunofluorescence Staining

Mice were anesthetized by 10% chloral hydrate intraperitoneally and transcardially perfused through the heart with saline followed by 4% paraformaldehyde in phosphate buffer (PBS, 0.1 M, pH 7.4). Brains were removed and immersed in the same fixative for 24 h. Then, the brains were transferred to 20% and 40% sucrose solution successively at 4°C. Therefore, tissue was sectioned on a freezing microtome (CM1950, Leica) at a thickness of 30 *μ*m in six regions and stored in 50% glycerin (diluted with PBS) at -20°C. The sections were removed from -20°C, washed with PBS for three times, and blocked with 5% BSA (containing 0.3% Triton-X100) for 1 h at room temperature. Whereafter, the sections were incubated with rabbit monoclonal anti-DCX (1 : 1000) or rabbit polyclonal anti-IL-1*β* (10 *μ*g/mL) and mouse monoclonal anti-GFAP (1 : 300) for 24 h at 4°C. After washing, the sections were incubated with Alexa Fluor 488 Goat anti-Rabbit IgG (1 : 1000) and Alexa Fluor 594 Rabbit anti-Goat IgG (1 : 2500) for 1 h in a dark room. After staining DAPI (4′,6-diamidino-2-phenylindole), images of positive staining in the hippocampus DG were captured using the LSM710 Confocal Laser Scanning Microscope (Zeiss, Germany).

### 2.8. Western Blot

The hippocampus was lysed in RIPA buffer (Beyotime, China) containing protease inhibitors and phosphatase inhibitors. The supernatant was collected, and protein concentration was determined by using a NanoDrop 2000 (Thermo, USA). An aliquot of 60 *μ*g of total protein was size-separated by 10% or 12% SDS-PAGE gels and was transferred to PVDF membranes. After blocking with 5% BSA in TBS containing 0.1% Tween-20 (TBST) for 1 hour, the membranes were incubated overnight at 4°C with the following primary antibodies: rabbit polyclonal anti-NLRP3 (1 : 1000), rabbit monoclonal anti-ASC (1 : 1000), mouse monoclonal anti-caspase-1 (1 : 300), rabbit polyclonal anti-IL-1*β* (0.5 *μ*g/mL), rabbit monoclonal anti-TXNIP (1 : 1000), rabbit monoclonal anti-DCX (1 : 1000), rabbit monoclonal anti-BDNF (1 : 1000), rabbit monoclonal anti-TrkB (1 : 1000), rabbit polyclonal anti-*β*-tubulin (1 : 3000), and rabbit polyclonal anti-GAPDH (1 : 3000). The membranes were then incubated with the secondary antibodies of horseradish peroxidase-conjugated antibodies for 1 hour at room temperature. The blots were visualized using the Chemistar™ High-sig ECL Western Blotting Substrate (Tanon, China). All experiments were performed in triplicate. Densitometric analysis was performed by using ImageJ software to compare band intensities between experimental conditions. Values calculated correspond to the ratio between the intensities of the gene of interest and *β*-tubulin or GAPDH followed by normalization to the control experimental condition (set to 1).

### 2.9. Statistical Analysis

Prism 6 software (GraphPad) was used for statistical analysis of data. Date are presented as means ± S.E.M. Differences between mean values were evaluated using one-way ANOVA followed by a post hoc LSD test. *p* < 0.05 was considered statistically significant.

## 3. Results

### 3.1. HPLC Analysis of MFX Aqueous Extract

**Figure 2:**
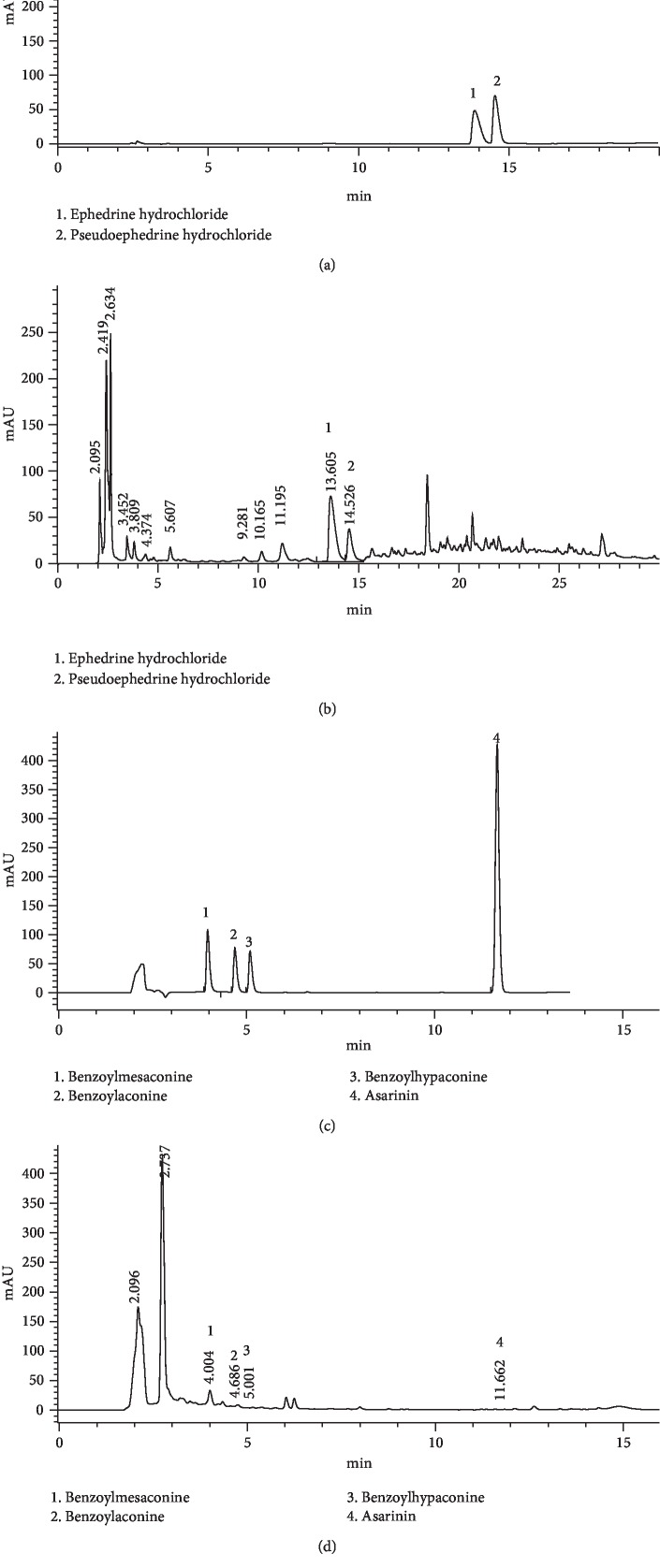
HPLC analysis of the MFX aqueous extract. (a) HPLC of the reference ephedrine hydrochloride and pseudoephedrine hydrochloride standard substances. (b) HPLC of the test MFX aqueous extract at 210 nm. (c) HPLC of the reference benzoylmesaconine, benzoylaconine, benzoylhypaconine, and asarinin standard substances. (d) HPLC of the test MFX aqueous extract at 235 nm.

The HPLC diagram for determination of standard content of ephedrine hydrochloride, pseudoephedrine hydrochloride, benzoylmesaconine, benzoylaconine, benzoylhypaconine, and asarinin was shown in Figures 2(a) and 2(c). Besides, the corresponding components of MFX were shown in Figures 2(b) and 2(c). The contents of main components of MFX were ephedrine hydrochloride (5.139 mg/g), pseudoephedrine hydrochloride (2.423 mg/g), and benzoylmesaconine (1.339 mg/g), respectively (Figures 2(b) and 2(d)). The results were in accordance with the provisions of the Chinese Pharmacopoeia (version 2015).

### 3.2. Acute LPS-Induced Depressive-Like Behavior in Mice Was Reversed by *Mahuang-Fuzi-Xixin* Decoction

**Figure 3:**
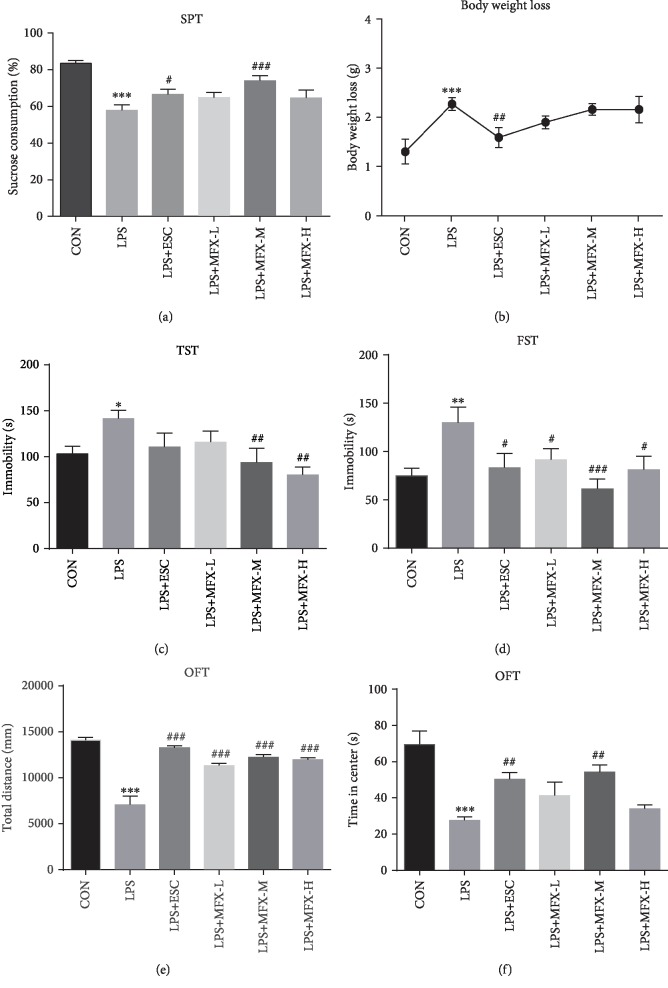
Effect of MFX treatment on body weight and depressive-like behavior in acute LPS-induced mice. (a) Sucrose consumption. (b) Body weight loss after LPS i.p. (c) Tail suspension test. (d) Forced swimming test. (e) The total distance traveled during the open field test. (f) The time spent in the central area during the open field test. The results were expressed as the mean ± S.E.M. (*n* = 6‐9). ^∗^*p* < 0.05, ^∗∗^*p* < 0.01, and ^∗∗∗^*p* < 0.001 compared to the control group. ^#^*p* < 0.05, ^##^*p* < 0.01, and ^###^*p* < 0.001 compared to the LPS group.

We exposed male Balb/C mice to acute LPS intraperitoneal injection to mimic depression to explore the potential mechanism that are modulated by MFX. As shown in Figure 3(a), LPS exposure reduced the sucrose consumption of mice (*F* = 9.370, *p* < 0.001), which was generally adopted as a common measure of anhedonia-like behavior in rodents [56]. MFX-M at a dose of 12.5 g/kg treatment significantly ameliorated this anhedonia in mice (*p* < 0.001). But 2.5 and 25 g/kg of MFX only had trends in SPT without statistical differences. Besides, mice treated with MFX-M consumed more sucrose than mice in the MFX-L group, indicating that the effects of MFX were dose-related.

Body weight was assessed at the 7^th^ day and 9^th^ day in order to observe the effect of LPS in mice. Mice treated with LPS exhibited a significant increase in body weight loss (Figure 3(b), *F* = 4.344, *p* = 0.0024), while MFX had no obvious effect on it.

The TST result showed that LPS led to a significant increase of immobility time (*F* = 2.965, *p* = 0.0228), and MFX (12.5, 25 g/kg) can reverse the immobility time prolonged by LPS (*p* < 0.01, Figure 3(c)).

As shown in Figure 3(d), the data from FST revealed that LPS exposure increased the immobility time in the behavioral despair model (*F* = 3.327, *p* = 0.0130), but it was reversed by MFX at the dose of 2.5, 12.5, and 25 g/kg (*p* < 0.05, *p* < 0.001, *p* < 0.05, respectively).

In order to rule out the effect of MFX on spontaneous activity in mice, OFT was tested in mice which were only given MFX (2.5, 12.5, and 25 g/kg) for 1 week. The three groups have no difference in the total distance traveled or central time (data not shown). Meanwhile, after intraperitoneal injection of LPS, the total distance during the OFT was significantly reduced in mice compared to normal mice (Figure 3(e), *F* = 25.98, *p* < 0.0001), as well as the time spent in the central area (Figure 3(f), *F* = 7.797, *p* < 0.0001). Therefore, MFX was effective in inducing antidepressant effect, and MFX-M at the dose of 12.5 g/kg was an effective dose and used in the following experiments.

### 3.3. NLRP3 Inflammasome Activation Was Inhibited by MFX Treatment in the Hippocampus of LPS-Induced Mice

**Figure 4:**
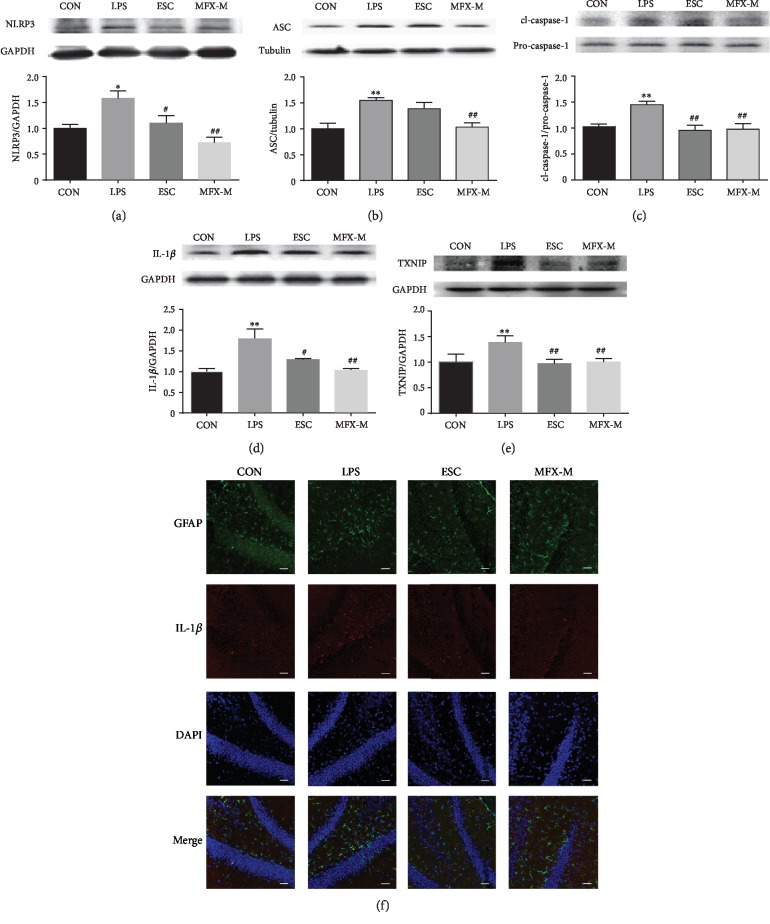
MFX-M alleviates the NLPR3 inflammasome activation in the hippocampus of LPS-stimulated mice. Representative blots and statistical graphs of relative protein expression of NLRP3 (a), ASC (b), cl-caspase-1, pro-caspase-1 (c), IL-1*β* (d), and TXNIP (e). (f) Frozen sections of brain tissue were subjected to immunofluorescence staining. The hippocampus sections were incubated with anti-GFAP antibody (green), anti-IL-1*β* (red), and DAPI (blue) and were observed by a laser scanning confocal microscope. Scale bar = 40 *μ*m. Data were the mean ± S.E.M. (*n* = 3 experimental replicates/group). ^∗^*p* < 0.05 and ^∗∗^*p* < 0.01 compared to the control group. ^#^*p* < 0.05 and ^##^*p* < 0.01 compared to the LPS group.

Neuroinflammation is thought to be fundamental in the etiology of MDD [57], and NLRP3 inflammasome is closely related to neuroinflammation. To test whether MFX could block NLRP3 activation, we examined the impact of MFX on NLRP3 inflammasome and IL-1*β* secretion, as well as TXNIP, which was closely related with the activation of NLRP3 inflammasome. MFX-M treatment suppressed LPS-induced caspase-1 activation (Figure 4(c), *F* = 6.896, *p* = 0.0131) and IL-1*β* (Figure 4(d), *F* = 7.896, *p* = 0.0089) production in the hippocampus, respectively. Moreover, MFX-M also reduced the expression of NLPR3 (Figure 4(a), *F* = 8.280, *p* = 0.0078) and ASC (Figure 4(b), *F* = 8.606, *p* = 0.0069). Besides, the levels of TXNIP in the hippocampus were markedly increased in LPS-induced mice, and treatment with MFX-M inhibited the increase in the TXNIP level (Figure 4(e), *F* = 8.748, *p* = 0.0066). The similar results were observed by immunofluorescence analysis in the dentate gyrus in LPS-treated mice as IL-1*β* was increasing. Besides, the GFAP, a biomarker of astrocyte, was activated in the dentate gyrus in LPS-induced mice, which means LPS stimulating the astrocyte activation. (Figure 4(f)). The results were consistent with the literature [58, 59]. Our results indicated that MFX-M specifically inhibited NLPR3 inflammasome activation.

### 3.4. LPS-Induced Hippocampal Neurogenesis Decrease in Mice Was Reversed by MFX Treatment

**Figure 5:**
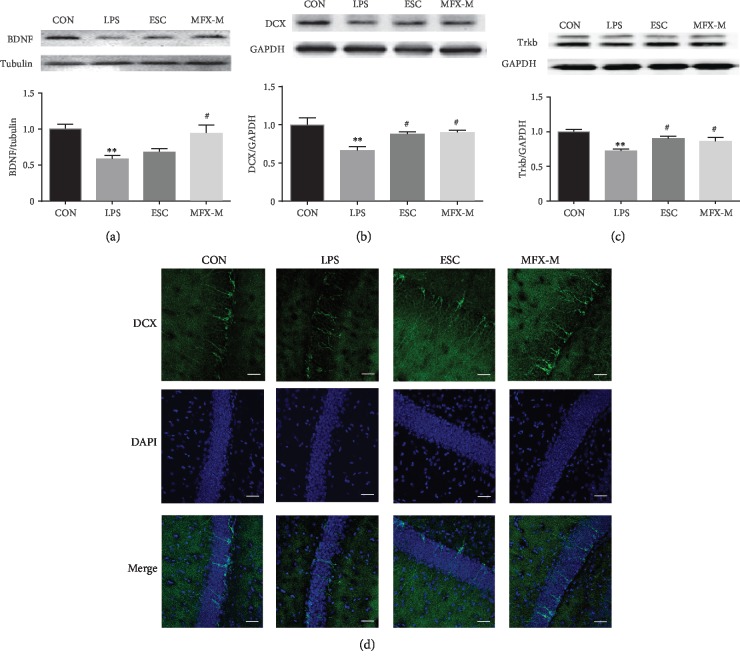
Effect of MFX-M on the BDNF and DCX levels in LPS-treated mice. Representative blots and statistical graphs of relative protein expression of BDNF (a), DCX (b), and TrkB (c). (d) Immunolabeling and confocal imaging analysis showed the DCX expression in the MFX-treated mice compared with LPS-induced mice. DCX (green) and DAPI (blue); immunofluorescent staining in dentate gyrus in mice. Scale bar = 40 *μ*m.

To study the possible influence of MFX on neurogenesis in LPS-treated mice, we compared the BDNF, DCX, and TrkB expression by using Western blot. After LPS injection, the BDNF protein displayed a lower level compared to the normal mice (Figure 5(a), *F* = 6.975, *p* = 0.0127). In parallel, LPS also decreased the expression of TrkB in the hippocampus of mice (Figure 5(c), *F* = 7.988, *p* = 0.0086). Consistent with the results, the DCX protein, a specific marker of immature neurons, was significantly attenuated in the hippocampus of LPS-treated mice (Figure 5(b), *F* = 6.154, *p* = 0.0179). Compared to the LPS-induced mice, pretreatment with MFX-M for 7 d, the expression of BDNF (*p* = 0.0108), TrkB (*p* = 0.0443), and DCX (*p* = 0.0183) was elevated obviously. Immunofluorescence staining also revealed that LPS significantly decreased the number of DCX-positive cells in the dentate gyrus, and MFX-M could reverse this change (Figure 5(d)).

## 4. Discussion

*Mahuang-Fuzi-Xixin* decoction is a commonly used traditional Chinese prescription which was first written by Zhang Zhong-jing. *Radix Aconiti Lateralis* polysaccharide and alkaloids are the main components of *Radix Aconiti Lateralis*. *Fuzi* polysaccharide-1 (FPS), a water-soluble polysaccharide isolated from *Fuzi*, could promote neurogenesis through the BDNF signaling pathway and reverse avoidance behavior by chronic defeat stress, then exert antidepressant effect [43]. Similarly, *Fuzi* total alkaloid could regulate the CREB-BDNF pathway in ovariectomized mice, which show the antidepressant-like effect [60]. Methyleugenol, as the main component of *Asarum*, produces antidepressive effect in rats forced to swim [61]. *β*-Asarone, another volatile oil of *Asarum*, has been proved to have antidepressant effect in chronic unpredictable mild stress (CUMS) mice by regulating neurogenesis through the ERK1/2-CREB signaling pathway [62]. In brief, the essential oil from *Asarum heterotropoide*s (EOAH) could inhibit depression-like behavioral responses [63]. Besides, sesamin, as a nonvolatile chemical constituent of *Asarum*, also improved CUMS-induced depression via inhibiting neuroinflammation [64]. In addition, *Ephedrae* is less reported on antidepressants. From the clinical application of MFX, MFX is widely used in respiratory and immune system diseases due to strong anti-inflammation and antianaphylaxis [35, 39, 65, 66]. Nevertheless, the impact on antidepressant is limited. Interestingly, the anti-inflammatory effect is the pharmacological effect of *Radix Aconiti Lateralis*, *Ephedrae*, and *Asarum*. In light of this, it is tempting to conjecture that MFX can be used for the treatment of MDD as MDD is closely related to inflammation.

Up to now, according to the Chinese Pharmacopoeia (version 2015), ephedrine hydrochloride, pseudoephedrine hydrochloride, benzoylmesaconine, benzoylaconine, benzoylhypaconine, and asarinin are the main compounds of *Radix Aconiti Lateralis*, *Ephedrae*, and *Asarum*, which can be used for quality control of MFX. In our study, HPLC was used to test the above components and the results were in accordance with the provisions of the Chinese Pharmacopoeia. We wonder if it could provide a basis for the subsequent development of a traditional Chinese medicine compound. Besides, benzoylaconine and benzoylhypaconine were considered to be part of the material bases of MFX on anti-inflammation and immunosuppression [67].

Bacterial lipopolysaccharide (LPS), which is a potent activator of the immune system, is usually used to study the inflammation-associated depression [54, 57, 68]. The present study revealed that MFX showed antidepressant effect in LPS-induced mice. Our results indicate that mice exhibited a typical depressive-like behavior after intraperitoneal injection of LPS for 24 h, such as reduced rearing times in the center and total distance traveled in OFT, decreased consumption for saccharose, and extended immobility in TST and FST. Meanwhile, the behavior deficits induced by LPS were reversed by MFX. Then, further studies are needed to clarify the mechanism between MFX and antidepressant effect. It should be noted that this study has examined the antidepressant effect of MFX only in the LPS-induced animal model; our findings need to be confirmed in other animal models like chronic unpredictable mild stress (CUMS) and social defeat.

Clinical studies involving patients with MDD have found increased gene expression of NLRP3 and caspase-1 in blood cells and increased serum levels of IL-1*β* and IL-18 in nontreated patients [17]. NLRP3 inflammasome, as the most extensively studied intracellular multiprotein complex [69], can be activated by a variety of dangerous signals like crystalline and particulate matter, extracellular ATP, pore-forming toxins, and RNA-DNA hybrids [70]. Activating NLRP3 inflammasome requires a two-step paradigm, whereby an initial signal facilitates the transcription of the NLRP3 protein, while subsequent activating stimuli remove physical and kinetic barriers to promote the step-wise assembly of the multimeric complex. Once activated, the cysteine caspase-1 of the NLRP3 inflammasome cleaves zymogen inflammatory cytokines pro-IL-1*β* and pro-iL-18 into their functional conformation [71]. We found that MFX treatment improved the LPS-induced inflammation observed in the hippocampus through regulating the activation of NLRP3 inflammasome and reducing the release of IL-1*β*. Besides, in our study, we found that MFX suppressed LPS-induced expression of TXNIP, and this is consistent with the studies [22]. However, the mechanism in MFX regulating NLRP3 inflammasome activation through TXNIP in this study is still unknown and would be studied deeper in the following experiment.

Besides, the current work provides evidence that both DCX and BDNF protein were enhanced in the hippocampus after MFX treatment in LPS-induced mice. BDNF is a nerve growth factor that has antidepressant-like effects in animals. Neurotrophic signaling is sensitive to activity of the inflammatory and stress response systems. In this context, our study has shown that intraperitoneal injection of LPS in mice could not only lead to neuroinflammation but also reduce the BDNF expression. After MFX treatment, BDNF protein could be upregulated. Meanwhile, MFX could increase the protein level of TrkB content of LPS-induced mice. Studies show that BDNF preferentially activates TrkB receptor in the developed brain [72, 73]. DCX is expressed by determined progenitor cells in the SGZ and is considered a specific marker of immature neurons [74]. Similar to the BDNF and TrkB proteins, LPS-induced mice showed downregulation in DCX protein, while MFX could reverse this change. Thus, our findings indicate that MFX could play an antidepressant role by regulating the TrkB/BDNF signaling pathway.

Interestingly, our present study showed that GFAP (a biomarker of astrocyte) was increased in the dentate gyrus in LPS-induced mice, which means LPS stimulating the astrocyte activation. As the most abundant cell type in the brain, the primary roles of astrocytes include the protection of neurons, release of neurotrophic factors, and participation in mediating and regulating neuroinflammation effects [75–77]. We wonder if the activation of inflammation and reduced neurogenesis in LPS-induced mice were associated with the astrocyte disturbance. Subsequent experiments will be performed on primary astrocytes.

Collectively, our findings suggested that MFX could alleviate the depressive-like symptoms induced by LPS, which might be related to the regulated TXNIP/NLRP3 pathway and TrkB/BDNF pathway in the hippocampus. Hence, our results probably provide an insight into the potential of traditional Chinese medicine in therapeutic intervention for depression. Despite this, it remains unclear about the mechanism in MFX regulating astrocytes. The effective material basis of MFX is still unclear. Further studies are warranted to capitalize on the protective effects of MFX on MDD in clinical practice.

## 5. Conclusion

1. Acquit lipopolysaccharide treatment induced depressive-like behaviors in mice
2. Mahuang-Fuzi-Xixin decoction (MFX) reversed LPS-induced depressive behaviors
3. MFX inhibit the NLRP3 inflammasome activation in the hippocampus of LPS-treated mice
4. MFX could promote the neurogenesis in the hippocampus of LPS-treated mice

## Data Availability

The data used to support the findings of this study are available from the corresponding author upon request.

## Supplementary Materials

Supplementary Materials The behavioral indexes and protein data used to support the findings of this study were available in the supplementary material.
