# Supplementary material for: Mahuang-Fuzi-Xixin Decoction Reverses Depression-Like Behavior in LPS-Induced Mice by Regulating NLRP3 Inflammasome and Neurogenesis
Source: Neural Plast. 2019 Nov 11;2019:1571392. doi: 10.1155/2019/1571392 (PMC6877957; doi:10.1155/2019/1571392)
Supplement: Supplementary Materials — The behavioral indexes and protein data used to support the findings of this study were available in the supplementary material. [file 1571392.f1.zip › behavior tests and blots.pptx]

## Slide 1
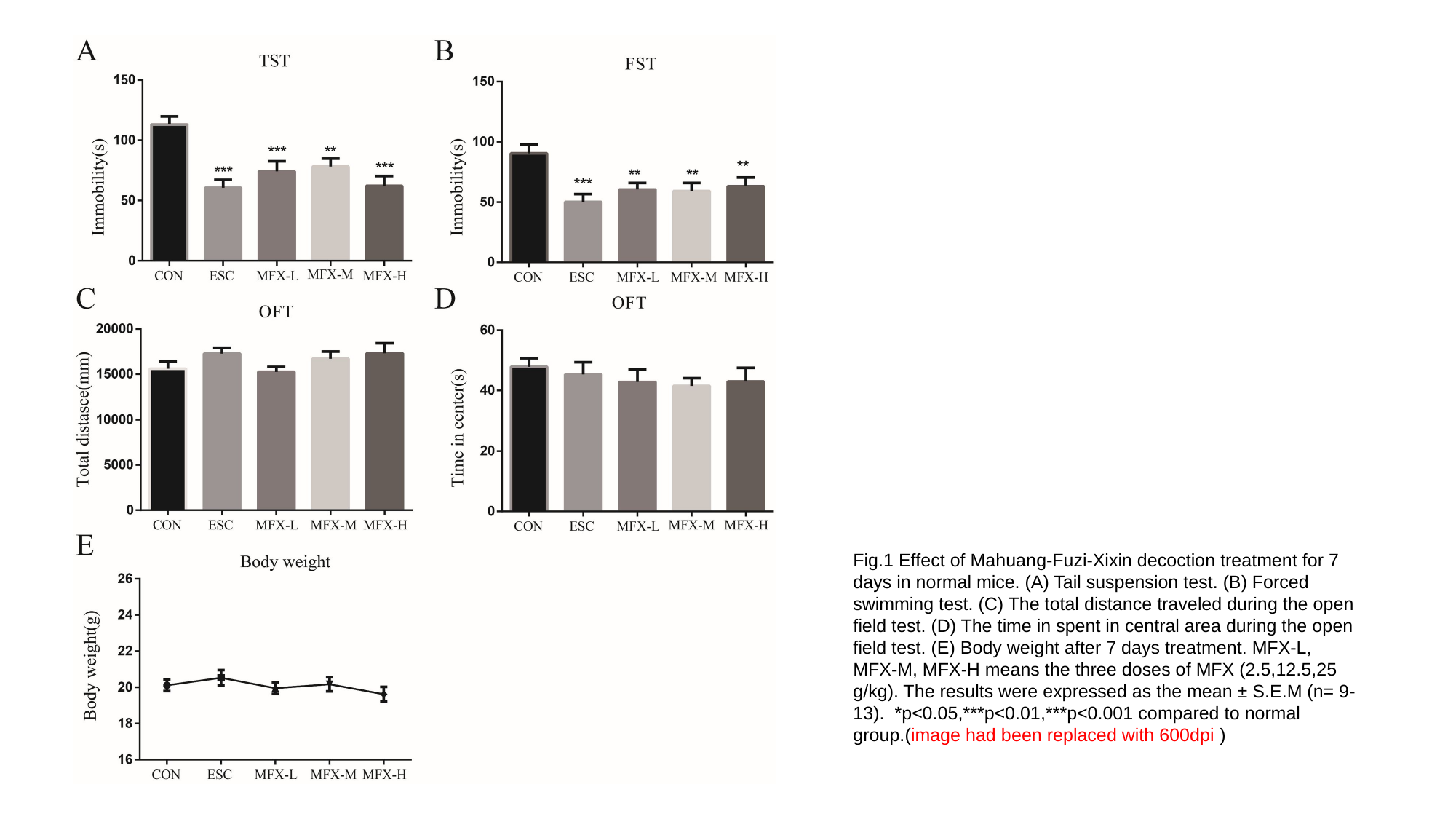

Fig.1 Effect of Mahuang-Fuzi-Xixin decoction treatment for 7 days in normal mice. (A) Tail suspension test. (B) Forced swimming test. (C) The total distance traveled during the open field test. (D) The time in spent in central area during the open field test. (E) Body weight after 7 days treatment. MFX-L, MFX-M, MFX-H means the three doses of MFX (2.5,12.5,25 g/kg). The results were expressed as the mean ± S.E.M (n= 9-13). *p<0.05,***p<0.01,***p<0.001 compared to normal group.(image had been replaced with 600dpi )

## Slide 2
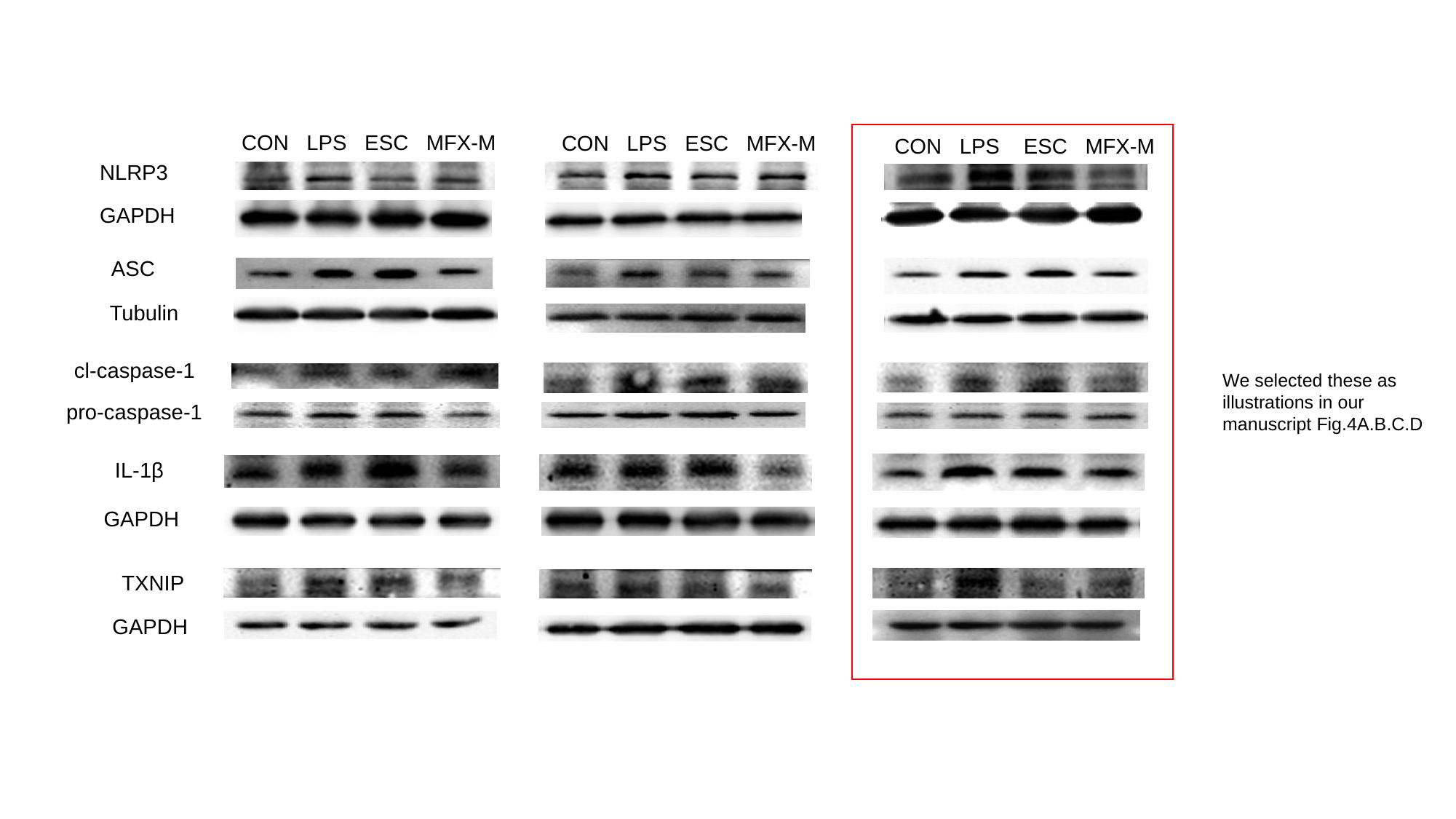

CON LPS ESC MFX-M
CON LPS ESC MFX-M
CON LPS ESC MFX-M
NLRP3
GAPDH
ASC
Tubulin
cl-caspase-1
We selected these as illustrations in our manuscript Fig.4A.B.C.D
pro-caspase-1
IL-1β
GAPDH
TXNIP
GAPDH

## Slide 3
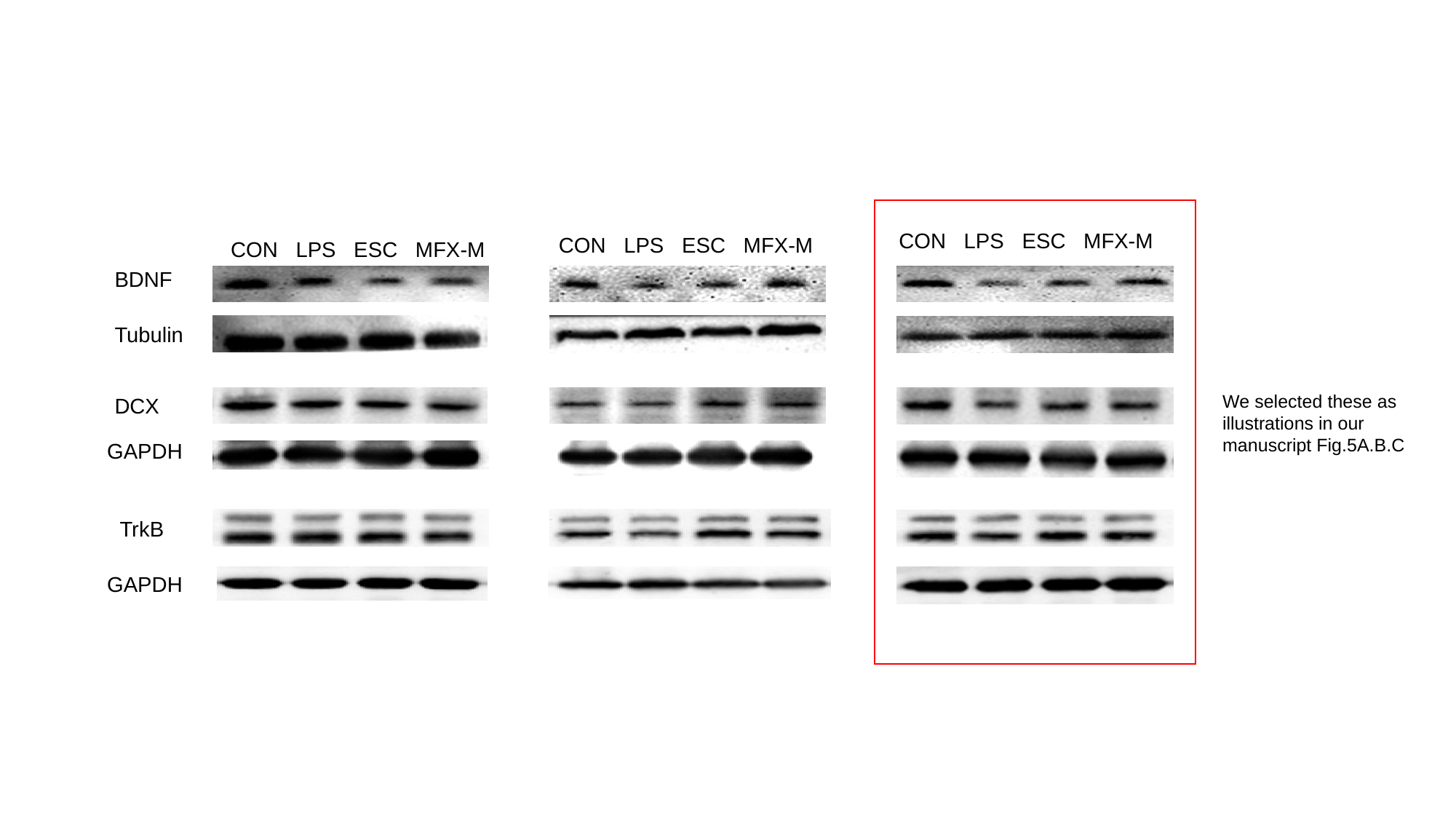

CON LPS ESC MFX-M
CON LPS ESC MFX-M
CON LPS ESC MFX-M
BDNF
Tubulin
We selected these as illustrations in our manuscript Fig.5A.B.C
DCX
GAPDH
TrkB
GAPDH
